# Advancing Skin Rejuvenation Through Ultrasound-Enhanced Non-Invasive Delivery of Hyaluronic Acid Nanoparticles

**DOI:** 10.3390/nano15221739

**Published:** 2025-11-18

**Authors:** Leah Shimonov, Chen Benafsha, Shir Harel, Ksenia M. Yegodayev, Uzi Hadad, Riki Goldbart, Tamar Traitel, Yuval Krieger, Moshe Elkabets, Joseph Kost

**Affiliations:** 1 Department of Chemical Engineering, Ben-Gurion University of the Negev, Beer-Sheva 8410501, Israel; leahshim@post.bgu.ac.il (L.S.); goldbart@post.bgu.ac.il (R.G.); tamluz@post.bgu.ac.il (T.T.); 2 The Shraga Segal Department of Microbiology, Immunology and Genetics, Faculty of Health Sciences, Ben-Gurion University of the Negev, Beer-Sheva 8410501, Israelmoshee@bgu.ac.il (M.E.); 3 The Ilse Katz Institute for Nanoscale Science and Technology, Ben-Gurion University of the Negev, Beer-Sheva 8410501, Israel; uzihad@bgu.ac.il; 4 Department of Plastic Surgery, Soroka Medical Center, Faculty of Health Sciences, Ben-Gurion University of the Negev, Beer-Sheva 8410501, Israel

**Keywords:** high molecular weight hyaluronic acid, modified starch, nanoparticles, residence time, low frequency ultrasound

## Abstract

High-molecular-weight hyaluronic acid (HMw-HA) is widely used for skin rejuvenation and anti-aging. However, its rapid degradation and <24 h cutaneous residence limit its therapeutic potential. Cross-linked HA fillers were developed to improve longevity, but their high viscosity and invasive administration may reduce biocompatibility and patient comfort. To overcome these challenges, we developed a biocompatible nanocarrier based on quaternized starch (Q-starch) that encapsulates linear HMw-HA into nanoparticles (NPs) of ~100 nm with a ζ-potential of ~−35 mV. These NPs retained spherical morphology over seven days without aggregation. The resulting HMw-HA-NPs significantly improved HA stability and extended its skin residence time compared to commercially available HA, while preserving its biological function to stimulate collagen production in murine models. To enable non-invasive delivery, we applied low-frequency ultrasound (LFUS), which transiently enhanced skin permeability, facilitated deeper NPs penetration, and further elevated collagen at day 14. Altogether, this strategy offers a promising needle-free alternative to traditional HA treatments by improving stability, skin penetration, and therapeutic efficacy. The combination of HMw-HA-NPs with LFUS addresses key limitations of current dermo-esthetic therapies and supports the development of patient-friendly skin rejuvenation technologies.

## 1. Introduction

Hyaluronic acid (HA) is a naturally occurring linear hydrophilic polymer consisting of disaccharide units of (β,1–4)-D-glucuronic acid-(β,1–3)-N-acetyl-D-glucosamine [1,2,3]. Approximately 50% of the HA produced by skin cells such as fibroblasts resides in the skin dermis and epidermis [1,2]. Some of HA’s functions in the skin are maintaining hydration and elasticity due to its capability of holding water molecules up to 1000 times its volume [4]. Moreover, HA is known to activate fibroblast cells, promoting collagen production through interactions with specific surface receptors such as CD44 and RHAMM [5,6,7,8]. These receptors initiate intracellular signaling pathways like PI3K/AKT and MAPK/ERK, which enhance collagen synthesis, tissue repair, and cell survival [7,8]. Collagen is produced inside fibroblasts and secreted into the extracellular space in the dermis layer, where it forms fibrils in the extracellular matrix (ECM), consequently providing structural support to the skin [9,10]. During the skin aging process, there is a significant decrease in HA synthesis and distribution that leads to a less resilient skin structure that contributes to the formation of fine lines and wrinkles [2,11,12]. Accordingly, the delivery of external HA for skin rejuvenation and wrinkle filling is of high significance in esthetic applications [12]. However, the following two major challenges hinder effective HA delivery into the skin: (1) its inability to penetrate the stratum corneum (SC), the outermost skin layer, which acts as a barrier permitting only small, lipophilic molecules of up to ~500 Da [13]; and (2) its rapid enzymatic degradation by hyaluronidases within the skin, resulting in a short half-life of less than 24 h [1,11,12,14].

To overcome the SC barrier, HA is commonly administered via intradermal or subcutaneous injections. To address the issue of rapid degradation, the HA chains are often chemically cross-linked to increase their longevity in the skin [14,15,16,17]. However, despite their efficacy, these injectable treatments pose several limitations. Cross-linked HA formulations, while more durable, often exhibit high viscosity, making them more difficult to inject and increasing the risk of uneven distribution and an unnatural appearance [16,18,19,20]. Furthermore, the chemical cross-linkers used to stabilize HA may trigger local side effects, such as hypersensitivity reactions, facial edema, nodules, and localized inflammation [21,22,23]. Even with linear HA used in skin boosters, patients often require multiple injections over large areas, resulting in significant discomfort and pain [16,24,25]. Although these formulations have received regulatory approval and are used globally [26], their invasiveness, pain, and potential side effects limit their desirability.

Non-injectable HA formulations, such as creams and serums, provide hydration at the skin surface but are unable to reach deeper layers due to the SC barrier, resulting in limited effectiveness for skin rejuvenation [27]. To overcome the SC barrier, minimally invasive approaches like microneedling, laser-assisted delivery, and thermal-based technologies have been explored [20,21]. However, these methods still involve skin disruption and have not demonstrated consistent or clinically meaningful deep dermal HA delivery.

Together, these limitations highlight the need for a novel, non-invasive, and effective strategy for delivering high-molecular-weight HA (HMw-HA) into the deeper layers of the skin.

Various carrier systems have been investigated to improve the dermal delivery of HA in esthetic applications, including lipid-based, polymeric, and polysaccharide-based approaches [16,24]. However, many of these systems still face limitations in terms of stability, efficiency, or compatibility with HMw-HA, emphasizing the need for alternative strategies.

**Figure 1:**
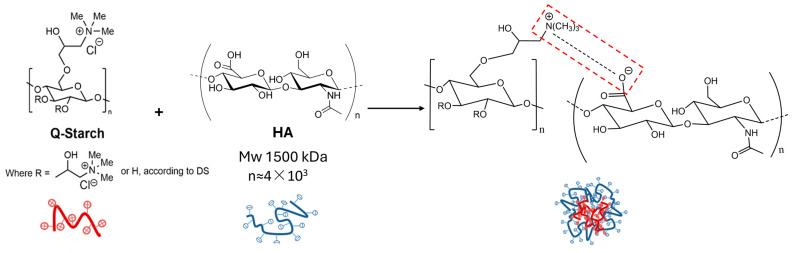
Schematic presentation of HMw-HA/Q-starch assembly.

In this study, we aimed to overcome these limitations by developing a novel, non-invasive delivery system for HMw-HA using a cationic polysaccharide derived from potato starch modified with quaternary amine groups (Q-starch) as a carrier. We hypothesized that complexing HMw-HA with a cationic quaternized starch (Q-starch) would yield nano-sized particles with enhanced dermal penetration and prolonged skin residence. By leveraging electrostatic interactions between the negatively charged HA and the positively charged Q-starch (Figure 1), this system may offer protection against enzymatic degradation while maintaining the intrinsic biocompatibility of polysaccharide-based carriers. To the best of our knowledge, this represents the first application of a Q-starch-based delivery system for topical HMw-HA in esthetic formulations. Q-starch has been previously explored in our lab as a versatile, non-toxic carrier [28,29,30], and its physicochemical properties make it a promising platform for the non-invasive delivery of large hydrophilic biomolecules like HMw-HA. To support non-invasive HMw-HA delivery, low-frequency ultrasound (LFUS) was considered as a skin permeation enhancer. LFUS has been shown to improve skin permeability and facilitate the transdermal delivery of both small and large molecules [31,32,33]. When combined with 1% sodium lauryl sulfate (SLS), LFUS exhibits a synergistic effect in enhancing skin penetration, as demonstrated in FDA-approved technologies such as SonoPrep^®^ [34,35]. This approach offers a safe and effective means to transiently disrupt the stratum corneum barrier without causing significant irritation.

This study presents a novel approach that combines Q-starch-based HMw-HA nanoparticles with ultrasound-enhanced delivery, aiming to develop an effective, non-invasive strategy for deep dermal delivery of HA in esthetic dermatology.

## 2. Materials and Methods

### 2.1. Materials

Sodium hyaluronate (HA, 1500 KDa, 026830) was purchased from Lifecore (Chaska, MN, USA). N-hydroxysulfosuccinimide sodium salt (Sulfo-NHS, P56485), N-(3-dimethylaminopropyl)-N-ethylcarbodiimide hydrochloride (EDAC, E1769), sodium chloride (NaCl, S-0399), 2-(N-morpholino) ethanesulfonic acid hydrate (MES hydrate, M2933), soluble starch (S-2630), sodium hydroxide (S-0399), 3-chloro-2-hydroxypropyl trimethyl ammonium chloride (CHPTAC, 348287), dialysis cellulose membrane (D9652), phosphate-buffered saline (PBS, P4417), and sodium lauryl sulfate (SLS, L5750) were purchased from Sigma-Aldrich (Rehovot, Israel). HiLyte^TM^ Fluor 488 amine, TFA salt, (81162), and Hylite Fluor 647 amine dye (AS-81162) were purchased from Anaspec (Fremont, CA, USA). Xylene (0313959), acetone (01030521), and ethanol (05250502) were purchased from Bio-Lab (Jerusalem, Israel). Paraformaldehyde (PFA) ampules 16% (BN15710) and Masson trichrome kit (04-010802BN) were purchased from Bar Naor Ltd. (Petah Tiqwa, Israel). Alcian Blue (pH 2.5) Stain Kit (VE-H-3501) was purchased from Zotal (Tel Aviv, Israel). DMEM medium (01-050-1A), fetal bovine serum (FBS, 04-121-1A), trypsin ethylenediaminetetraacetic acid (EDTA, 03-052-1B), L-glutamine (03-020-1B), penicillin–streptomycin (03-031-1B), and trypan blue (03-102-1B) were purchased from Biological Industries (Beit Ha’emek, Israel). Goat Anti-Type I Collagen-AF488 (1310-30) was purchased from Enco (Petah Tiqwa, Israel). Prolong gold antifade reagent with DAPI (P36935) and NucBlue^TM^ Live Ready Probes^TM^ reagent (R37605) were purchased from Invitrogen (Carlsbad, CA, USA). Full-thick skin from porcine ears was purchased from the Institute of Animal Research (Lahav, Israel).

### 2.2. HMw-HA Labeling

HMw-HA was fluorescently labeled using carbodiimide chemistry as described by Sapir Y. et al. [36]. Briefly, 20 mg of 1500 KDa sodium hyaluronate was dissolved in DDW (0.2% *w*/*v*) with MES buffer and NaCl (pH 6.5). EDAC and sulfo-NHS were added for carboxylic group activation and stabilization. After 3 h of stirring, Hylite Fluor 647 amine dye was added and incubated for 12 h. The product was purified via dialysis (Mw 11 KDa) against DDW for three days, lyophilized, and stored at 4 °C.

### 2.3. Starch Quaternization

Low-molecular-weight potato starch was quaternized following the method of Amar Lewis et al. [29]. Briefly, a starch solution (500 mg in NaOH, 0.19 g mL^−1^) was stirred at room temperature (RT) for 30 min. CHPTAC was prepared in DDW (0.32 g mL^−1^) and added, and the reaction was stirred for 20 h at RT. The product was precipitated using an acidified ethanol–acetone mixture (1:3), washed, dissolved, dialyzed (14 KDa Mw), and lyophilized for 72 h.

### 2.4. Q-Starch Labeling

Q-starch was labeled with 5-(4,6-dichlorotriazinyl) amino fluorescein (5-DTAF). A 100 mg Q-starch solution (3 mL DDW) was adjusted to pH 11–12 with NaOH and stirred for 30 min. 5-DTAF (7.5 mg in DMSO) was added and stirred for 24 h in the dark. The reaction was neutralized with HCl, dialyzed (11 KDa Mw) against PBS and DDW, and lyophilized to obtain purified Q-starch^5-DTAF^.

### 2.5. HMw-HA-NPs Formation

HMw-HA-NPs were formed at varying N/O molar ratios, representing the amine groups on Q-starch (N) to the carboxylic groups on HA (O). HA moles were calculated using its mass and monomer molecular weight (Equation (1)), while Q-starch quantities were determined to match the desired molar ratio (Equation (2)). HA (1 mg mL^−1^) and Q-starch (0.4 mg mL^−1^) solutions were mixed with fixed HA volume (10 µL) and varying Q-starch volumes. The mixture was gently vortexed and incubated for 40 min at RT for self-assembly.

Equation (1) is as follows:

*O* = *X*/*M*_W_ (1)

where *O* is the moles of negative charge on the HA backbone (mmol), *X* is the amount of HA (mg), and *M_W_* is the molecular weight of one monomer HA (mg/mmol).

Equation (2) is as follows:

*N* (g of *Q-Starch*) = (*N*/*O*) × *O* × *M_W_*(*N*) × 100/%*N*(*g*
*N*/*g*
*Q-Starch*) (2)

where *N* is the moles of positive charge on the *Q-starch* backbone (mol), *O* is the moles of negative charge on the HA backbone (mol), and *M_W_*(*N*) is the molecular weight of nitrogen (g/mol).

### 2.6. ζ-Potential Measurements

The surface charge of HMw-HA-NPs was measured using a Zetasizer (ZN-NanoSizer, Malvern, UK) at 25 °C. NPs containing 26 mM HA were analyzed in U-tube cuvettes (DTS1070, Malvern, 1 mL volume) using the Smoluchowski model.

### 2.7. Size Characterization

The size of HMw-HA-NPs was measured using a NanoSight NS300 system (Malvern Instruments, Malvern, UK) with a 642 nm laser and 650 nm longpass filter to determine the diameter of HMw-HA-NPs in water solution. HMw-HA-NPs containing 13 mM HA were analyzed at RT in a flow cell (software: NTA 3.1(iss2)), with 50 s video clips captured under a 20× objective.

### 2.8. Cryogenic Transmission Electron Microscopy (Cryo-TEM) Analysis

HMw-HA-NPs size and morphology were visualized using cryo-TEM. Samples of 260 mM HA and 0.28 mg mL^−1^ Q-starch were vitrified on carbon lacey films by plunging into liquid ethane. The specimens were imaged at −178 °C using an FEI Tecnai 12 G2 TWIN TEM at 120 kV with a Gatan CCD camera (Gatan, Pleasanton, CA, USA).

### 2.9. Stability Evaluation of HMw-HA-NPs

To assess the physical stability of HMw-HA-NPs in water over time, freshly prepared NPs (260 mM HA, N/O = 0.25) were stored in aqueous solution at RT and analyzed at multiple time points (zero, one, three, and seven days post-preparation). Particle size distribution was measured by NanoSight NS300 (Malvern Instruments, UK) as described in Section 2.7, and ζ-potential was evaluated using the Zetasizer (Malvern Instruments, UK) as described in Section 2.6. In addition, viscosity measurements of the HMw-HA-NPs solution were performed using an Anton Parr MCR702 rheometer (Graz, Austria), equipped with a CCC27/MD/PTD/TS measuring cup and an ME27-4.635-40 cylinder geometry. Temperature was precisely controlled using an advanced Peltier system. The experimental shear rate range was 1–1000 s^−1^. Morphological evaluation of particle structure was performed by cryo-TEM (see Section 2.8) to qualitatively assess potential aggregation or structural collapse. All measurements were compared to freshly prepared samples to determine relative changes and assess long-term colloidal and rheological stability.

### 2.10. Effect of HMw-HA-NPs on Cells Culture of Human Dermal Fibroblasts

#### 2.10.1. Cell Line

Human dermal fibroblasts (BJs) were cultured in DMEM with 10% FBS and 1% penicillin–streptomycin in a 25 cm^2^ flask at 37 °C and 5% CO_2_. Cells were split every three days using 1 mL trypsin-EDTA and counted with a Countess™ II FL Automated Cell Counter (Carlsbad, CA, USA). Cell seeding utilized the Trypan blue exclusion method. All experiments were conducted in a sterile laminar flow hood sanitized with UV light and ethanol.

#### 2.10.2. Viability Assay

Fibroblasts (5 × 10^4^ cells/well) were seeded in 24-well plates and incubated for 24 h to reach ~50% confluence. Cells were treated with HMw-HA solution (0.1 mg mL^−1^), HMw-HA-NPs (260 mM HA), or media-only control for 72 h. After treatment, cells were washed, fixed with 4% PFA, and stained with crystal violet (CV). CV was dissolved in acetic acid, and absorbance was measured at 570 nm using a microplate reader (Infinite M200, Tecan Plate Readers, Männedorf, Switzerland). Results were normalized to control absorbance to indicate relative cellular viability, where higher absorbance corresponded with higher cellular viability.

#### 2.10.3. Cellular Uptake

Cellular uptake of fluorescent HMw-HA-NPs (N/O = 0.25, 260 mM HA) was analyzed by confocal microscopy. Confocal images were obtained by using an LSM880 inverted laser scanning confocal microscope (Carl Zeiss AG, Oberkochen, Germany) equipped with an Airyscan high-resolution detection unit and Plan-Apochromat 20×/0.8 (WD = 0.55 mm) M27 objective with a 488 nm laser. Parameters were set to avoid pixel intensity saturation and to ensure Nyquist sampling in the x–y plane. Fibroblasts (5 × 10^4^ cells/well) were cultured on glass bottom 8-well plates for 24 h. Cells were incubated with 80 µL NPs diluted to 400 µL with media for 24 h. After incubation, cells were stained with NucBlue for nuclear visualization for 20 min and washed with PBS. Fresh pre-warm media were added to the cells, and imaging was performed to capture representative locations in each well.

#### 2.10.4. HMw-HA-NPs Effect on Collagen Production in BJs

Fibroblast cells were seeded on cover glasses in 12-well plates and incubated for 24 h. The medium was then replaced with 850 µL of fresh media containing one of the following three groups: 150 µL of free HMw-HA solution (0.1 mg mL^−1^), 150 µL of HMw-HA-NPs (N/O 0.25, 0.1 mg mL^−1^ HA), or 150 µL of DDW as a control. After 72 h at 37 °C, cells were washed with PBS and fixed with 4% PFA for 30 min at RT. Following additional PBS washes, cells were permeabilized with 0.1% Triton X-100 in PBS and blocked using 5% BSA in PBS-T for 1 h. Subsequently, cells were incubated overnight at 4 °C with Alexa Fluor-488 anti-type 1 collagen antibody (1:400) for collagen detection. After final PBS washes, cells were mounted using DAPI Fluoromount-G^®^ for nuclear visualization. Fluorescence imaging was performed using excitation at 488 nm and emission at 496 nm to observe collagen. Fluorescence intensity was quantified using ImageJ software v0.4.0, and statistical analysis was conducted on triplicates with Prism software to evaluate significant differences among treatment groups.

#### 2.10.5. Statistical Analysis

Statistical analysis was performed using GraphPad Prism software (version 7) and presented as the mean ± SEM. For comparisons among three groups, *p* values were calculated by one-way Anova test. For comparisons among three groups split by two independent variables, *p* values were calculated by two-way Anova. *p* values of 0.05 (*), 0.01 (**), 0.001 (***), and 0.0001 (****) were considered statistically significant.

### 2.11. In Vivo Experiments

#### 2.11.1. Animals Care

All animal experiments were conducted using 9–12-week-old C57BL wild-type mice (Envigo) and 12–15-week-old Skh-1 hairless mice (Charles River). Skh-1 hairless mice were bred at our university. The experiments were carried out under the Institutional Animal Care and Use Committee (IACUC) of Ben-Gurion University of the Negev (BGU’s IACUC) according to specified protocols aimed at ensuring animal welfare and reducing suffering. The animal ethical clearance protocol numbers used for this study are IL-14-02-2020 and IL-83-11-2023.

#### 2.11.2. HMw-HA-NPs Safety Study

C57BL wild-type mice were anesthetized with xylazine and ketamine, and the hair on their backs was shaved prior to the experiment. A 25 µL dose of HMw-HA-NPs (260 mM concentration HA, HA labeled with Hylite Fluor 647 and Q-starch labeled with 5-DTAF) was injected into the dermis/subcutaneous layer using a 31G needle. The mice’s weight and vital signs were monitored daily for one week. Afterward, the mice were sacrificed, and major organs (heart, lungs, liver, spleen, and kidneys) were harvested. Fluorescence imaging of the organs was conducted using an optical imaging system (Newton 7.0 FT500, Vilber, Marne-la-Vallée, France) under the following parameters: excitation at 649 nm, emission at 674 nm, 100 ms exposure time, and a 20 × 20 cm field of view. Fluorescence signal intensity from the organs was quantified using Quant analysis software.

#### 2.11.3. HMw-HA-NPs Stability (HA Residence Time) and Collagen Production

C57BL wild-type mice were anesthetized with a combination of xylazine and ketamine, and 25 µL of HMw-HA-NPs (260 mM concentration) was injected into the dermis/subcutaneous layer following the shaving of their backs (using an electric clipper). The injection sites were marked, and twenty mice were divided into the following three groups: Group I consisted of four untreated control mice; Group II included eight mice injected with free HA in two areas on their backs; and Group III comprised eight mice injected with HMw-HA-NPs in two areas on their backs.

After 14 and 30 days, the mice were sacrificed, and skin sections approximately 1 cm around the marked injection sites were collected. Skin samples were fixed in 4% PFA, dehydrated, embedded in paraffin, and sectioned into 5 µm slices using a Leica RM2255 microtome. Slides underwent sequential washing with xylene (twice for 10 min), 100% ethanol (twice for 10 min), and progressively decreasing ethanol concentrations (95%, 70%, and 50%, each for 5 min), followed by distilled water and PBS washes (twice for 10 min each). Finally, the slides were stained with Alcian Blue (pH 2.5) for HA visualization and Masson trichrome for collagen visualization, following the manufacturer’s protocols.

#### 2.11.4. Skin Electrical Conductivity Measurements

Conductivity measurements were used to evaluate the permeability of the skin during US exposure. One electrode (Ag/AgCl_4_ mm disk electrodes, In Vivo Materia, Somerville, MA, USA) was positioned on the skin surface and the other at the incision that was made near the tail. A voltage of 50 mVpp and 10 Hz was applied using a function generator (Agilent 33120A, Palo Alto, CA, USA). The current was measured with a multimeter (Fluke 45 display multimeter; Fluke Corp., Everett, WA, USA).

#### 2.11.5. Permeability Experiments Following US Pre-Treatment

Permeability of HMw-HA-NPs was evaluated using Skh-1 hairless mice. Mice were anesthetized using a xylazine and ketamine combination, ensuring complete immobility during US exposure. Skin integrity was confirmed by measuring conductivity and defective skin was excluded from the study if conductivity exceeded 0.65 (kΩ·cm^2^)^−1^. A plastic chamber with a rubber ring (8 ± 2 mm in diameter) was secured to the back skin using Scotch super glue. The chamber was filled with 2 mL of 1% SLS in PBS, and a US probe was positioned within the chamber, 8 mm from the skin surface. US was applied as a pre-treatment using a Q-SONICA Q700 system (20 kHz, 12 W (cm^2^)^−1^), 1.3 cm probe diameter) in a 50% duty cycle (1 s on, 1 s off) to minimize thermal effects. Conductivity measurements were taken every 20 sec, with US application terminated upon reaching a plateau or 0.70 (kΩ·cm^2^)^−1^ conductivity, typically within 80–120 s. Post-US, the skin was washed with PBS, and HMw-HA-NPs (Q-starch^5-DTAF^, 260 mM) were applied within the chamber for 24 h, secured with a custom 3D-printed cover.

This experiment included the following three groups of mice (n = 4 per group): untreated mice (naïve), mice receiving US pre-treatment followed by HMw-HA-NPs, and mice receiving only HMw-HA-NPs without US. After 24 h, the mice were sacrificed, and skin samples were collected, processed, sectioned at 5 µm, and stained with DAPI for histological evaluation of NPs penetration.

#### 2.11.6. Biological Evaluation of Collagen Stimulation Post-Permeability Evaluation

To assess the biological effect of HMw-HA-NPs delivery, particularly on collagen production, a second in vivo experiment was conducted using the same mouse model. Mice were divided into the following three groups (n = 4 per group): naïve untreated mice, mice receiving US pre-treatment followed by topical application of HMw-HA-NPs for 14 consecutive days (Q-starch labeled with 5-DTAF), and mice treated with US only without NPs application. After 14 days of treatment, the mice were sacrificed, and skin tissues were collected and processed similarly to the first experiment. Tissue sections were stained using Masson’s trichrome to visualize collagen deposition and assess structural changes in the dermis following long-term exposure to the NPs.

## 3. Results and Discussion

### 3.1. HMw-HA-NPs Formation and Characterization

The aim of this study was to evaluate the formation, stability, and therapeutic potential of HMw-HA-NPs created through electrostatic interactions between negatively charged HMw-HA and a positively charged, modified Q-starch carrier. The Q-starch carrier serves the following two principal roles: (1) condensing the HMw-HA into nano-sized particles to enhance its skin penetration and (2) protecting HMw-HA from rapid degradation, thereby enabling sustained release and prolonged retention of HA within the skin.

The Q-starch carrier was synthesized by quaternizing natural starch, a process that introduces quaternary amine groups (positively charged) onto the starch backbone. Successful quaternization was confirmed by 1H-NMR, elemental analysis, and FT-IR spectroscopy, in accordance with established protocols and results from previous studies [29,37].

**Figure 2:**
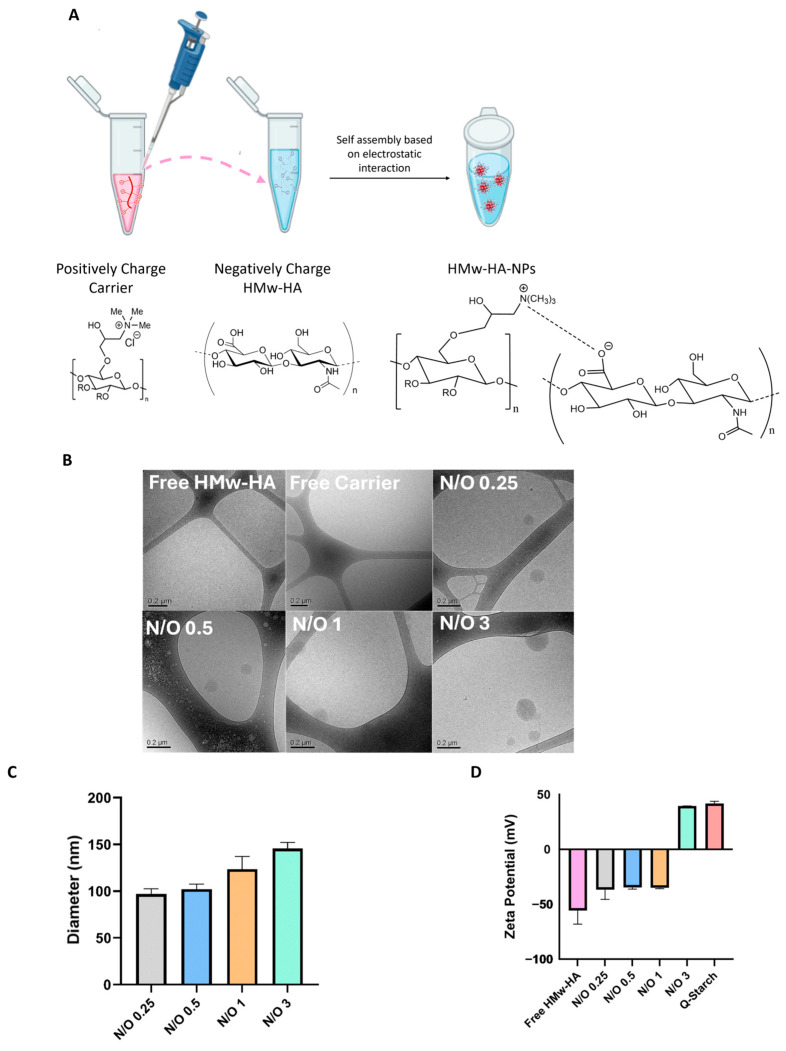
(**A**) Schematic illustration of HMw-HA-NPs preparation by self-assembly (created by Biorender). (**B**) Representative cryo-TEM images of freshly prepared HMw-HA-NPs at different N/O molar ratios (0.5 to 3), free HMw-HA, and free Q-starch. (**C**) HMw-HA-NPs diameter by NanoSight (average ± SEM, n = 3) at increasing N/O molar ratios. (**D**) Surface charge (ζ-potential) of free Q-starch, free HMw-HA, and HMw-HA-NPs at increasing N/O molar ratio (average ± SEM, n = 3).

To confirm the formation of HMw-HA-NPs and characterize their structure, a series of analytical methods were used, as illustrated schematically in Figure 1 and Figure 2A, which demonstrate the formation of HMw-HA-NPs via electrostatic interactions. Cryo-TEM imaging was used to determine whether NPs were formed, both in terms of size and shape. NPs of Q-starch and HMw-HA were prepared at specific N/O molar ratios of 0.25, 0.5, 1, and 3. Cryo-TEM representative images in Figure 2B demonstrated mostly small (approximately 100 nm in diameter), globular, and condensed NPs at all tested N/O ratios. Cryo-TEM was unable to clearly visualize free HMw-HA or free Q-starch, as both appeared completely clean on the grids (Figure 2B). This observation can be explained by the fact that linear HMw-HA and free Q-starch are well dissolved in aqueous solution and have low electron density, which makes them undetectable by cryo-TEM under these conditions. Importantly, no substantial morphological differences were observed between the NPs at different N/O ratios, possibly due to their similar compact structure and uniform condensation behavior, despite differences in size. The diameter of HMw-HA-NPs was also evaluated by NanoSight, as shown in Figure 2C. All measured diameters ranged between 100 and 140 nm, and the increase in the N/O molar ratio resulted in larger HMw-HA-NPs, probably due to the addition of Q-starch chains. Complementary DLS showed PDI = 0.356 for N/O = 0.25 and PDI = 0.0941 for N/O = 0.5, indicating moderate and narrow size distributions, respectively. These findings are consistent with the NanoSight profiles and cryo-TEM morphology, with no evidence of aggregation. To determine the surface charge of HMw-HA-NPs at different N/O molar ratios, ζ-potential measurements were performed. Free HMw-HA at a physiological pH is negatively charged due to the carboxylic groups on its backbone, presenting a negative ζ-potential of −70 mV, as can be seen in Figure 2D. The ζ-potential values of the NPs increased from negative values of ∼−36 mV for N/O of 0.25 to positive values of ∼40 mV at N/O of 3. Unexpectedly, up to a ratio of N/O 1, there was no significant change in the values of ζ-potential. This phenomenon likely occurs due to the spatial arrangement and distribution of the charged groups on the HMw-HA-NPs surface, where the negative groups of HMw-HA may dominate the surface and are not all associated with the positive groups on Q-starch carrier, even after adding additional Q-starch chains. The free Q-starch provided a highly positive ζ-potential value of 42 mV, confirming the starch substitution with quaternary amine groups. Furthermore, ζ-potential values greater than 30 mV or lower than −30 mV serve as a reliable indication of stable particles [38]. It is important to note that stable NPs were observed across all N/O molar ratios investigated, implying the probability of an extended lifetime of HA within the skin during treatment.

Following HMw-HA-NPs characterization, an N/O molar ratio of 0.25 was selected as the optimal ratio for further evaluation. This selection was based on the following three factors: 1. the amount of the carrier required at this ratio is minimal, 2. the NPs size is the smallest, and 3. the negative surface charge of the NPs, which resembles the negative charge of HA and probably promotes the bioavailability of complexed HMw-HA.

### 3.2. Physical Stability Evaluation of HMw-HA-NPs in Aqueous Suspension over Time

**Figure 3:**
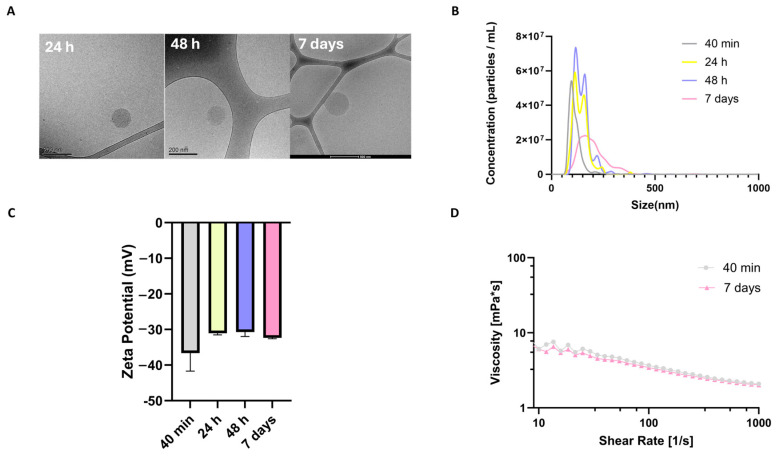
Physicochemical stability of HMw-HA-NPs in DDW over time: (**A**) cryo-TEM images of HMw-HA-NPs at 24 h, 48 h, and 7 days post-preparation; (**B**) particle size distribution measured by NanoSight at 40 min, 24 h, 48 h, and 7 days post-preparation; (**C**) ζ-potential measurements of HMw-HA-NPs at corresponding time points (average ± SEM, n = 3); (**D**) rheological measurements of viscosity profiles of HMw-HA-NPs at 40 min and 7 days under increasing shear rate.

Assessing the long-term physical stability of HMw-HA-NPs in aqueous phase is essential to ensure their structural integrity and therapeutic efficacy, as changes in particle size or morphology may compromise skin penetration capability. To this end, the stability of HMw-HA-NPs in ultrapure water (UPW) was evaluated under static storage conditions at RT (~25 °C) in closed vials for up to seven days. Cryo-TEM images (Figure 3A) revealed that the NPs retained their spherical morphology throughout the course of time. A gradual increase in particle diameter was observed between 24 h and 7 days, which is consistent with the hygroscopic nature of HA, known to absorb moisture and swell in aqueous environments. Importantly, no aggregation or fusion between NPs was detected, indicating uniform swelling while preserving nanoscale architecture.

This morphological trend was further supported by NanoSight particle size analysis (Figure 3B), which showed a gradual increase in particle size over time. At early time points (40 min and 24 h), the size distribution displayed more than one peak, indicating the presence of different particle populations. By seven days, the distribution curve broadened and shifted toward larger sizes, consistent with particle swelling. Notably, although particle size increased, the formulation did not exhibit aggregation, and the particles remained evenly dispersed in solution. This controlled size expansion may facilitate improved hydration potential and skin retention in vivo.

Measuring the surface charge of hydrated HMw-HA-NPs over time is critical, as it reflects their physical stability in suspension and provides insight into potential aggregation behavior. As can be seen in Figure 3C, across all samples, the ζ-potential remained consistently negative, ranging from −35.8 mV to −32.1 mV, indicating sufficient electrostatic repulsion between particles, minimizing the likelihood of aggregation or sedimentation over time. The stable surface charge further supports the structural integrity and dispersion of HMw-HA-NPs during storage.

Maintaining consistent viscosity is particularly important for topical or injectable formulations, as it ensures predictable application performance and dosing accuracy. Therefore, a rheological analysis was performed to evaluate whether the swelling of HMw-HA-NPs during storage affects their flow behavior (Figure 3D). Complementing the morphological and colloidal data, the results showed that viscosity and shear-thinning behavior were preserved even after seven days. Our aim here was to verify the time invariance of flow behavior rather than to establish an absolute viscosity value; the overlapping flow curves over 10–1000 s^−1^ indicate no significant degradation or aggregation affecting rheology between 40 min and day 7, in agreement with our independent cryo-TEM, NanoSight, and ζ-potential results showing no aggregation. The comparable flow curves between freshly prepared and day 7 formulations suggest that the internal structural organization of the NPs remains intact and is not disrupted by the hydration process. These results demonstrate a dual advantage: HMw-HA-NPs retain their stability in terms of size, surface charge, and viscosity over time, while also exhibiting controlled swelling behavior. This combination is particularly beneficial for dermal applications, as it enables gradual hydration and enhanced interaction with skin tissue during extended residence, potentially enhancing both the efficacy and reliability of topical or injectable formulations in clinical and esthetic settings.

### 3.3. Safety, Cellular Uptake and Collagen Synthesis of HMw-HA-NPs In Vitro

Given HA’s role in collagen production, we investigated the biological activity of HMw-HA when complexed with a Q-starch carrier, focusing on its safety, cellular uptake, and ability to stimulate collagen production in fibroblast cells (skin cells found in connective tissue, primarily responsible for producing and secreting components of the extracellular matrix such as collagen, elastin, and proteoglycans).

**Figure 4:**
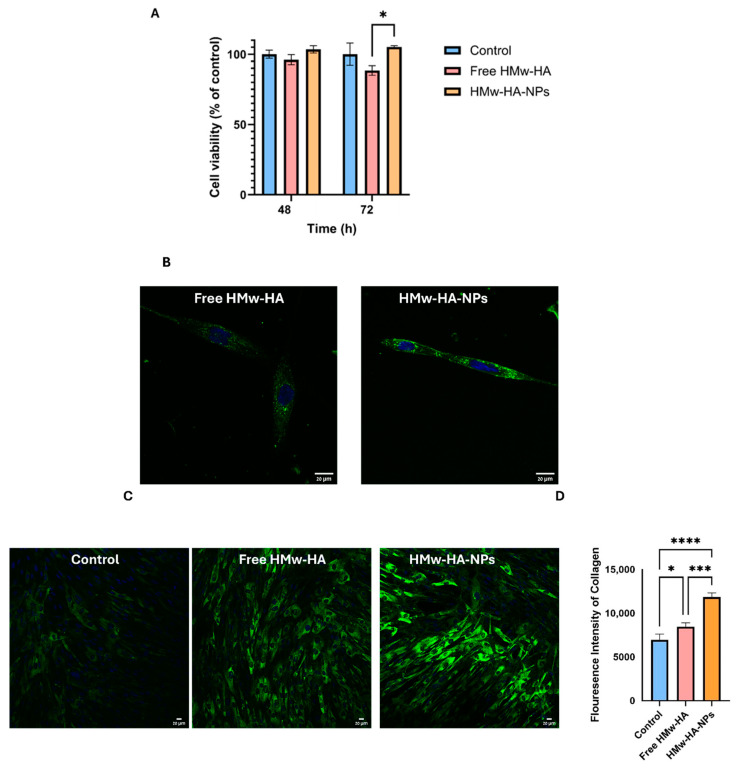
(**A**) Viability expression of fibroblast cells exposed to DMEM + 10% FBS 48 and 72 h post-treatment. The results are normalized to the control group (untreated cells). Free HMw-HA solution (0.1 mg mL^−1^) and HMw-HA-NPs at HMw-HA concentration of 260 mM. (* *p* < 0.05, average + SEM (n = 3)). (**B**) Representative live confocal microscopy images of fibroblast cells exposed to free HMw-HA^Hylite Fluor 488^ (0.1 mg mL^−1^) and HMw-HA-NPs (N/O = 0.25) for 24 h. HMw-HA is labeled in green, and the cell’s nucleus is labeled in blue by NucBlue. Bar: 20 µm. (**C**) Representative confocal microscopy images of immunofluorescence staining of collagen (stained in green) in cultures of fibroblast on glass 72 h after treatment with the following groups: DMEM + 10% FBS (control cells); free HMw-HA solution (0.1 mg mL^−1^); and HMw-HA-NPs. Bar: 20 µm. (**D**) Fluorescence intensity measurements of collagen in fibroblast cells exposed to different groups over 72 h, analyzed using ImageJ software. (* *p* < 0.05, *** *p* < 0.001, and **** *p* < 0.0001). Average ± SEM (n = 3).

The safety of HMw-HA-NPs was evaluated by measuring cellular viability after incubation with the HMw-HA-NPs for up to 72 h. We used a single application-relevant dose (0.1 mg mL^−1^) to ensure reproducible handling and exposure. Viability was assessed by the CV assay, chosen because it directly quantifies the number of adherent cells after washing, providing a conservative proxy for proliferation in adherent cultures. This approach is also well supported in the literature [39,40]. As demonstrated in Figure 4A, all the cell groups exposed to the NPs exhibited the same cellular viability as the unexposed control group, revealing no statistical differences between the different experimental groups after 72 h of exposure. At 72 h, however, a statistically significant difference was observed between the HMw-HA-NPs and free HMw-HA, which may imply that free HMw-HA at that concentration is slightly toxic to the cells. Figure 4B shows representative live confocal images of fibroblast cells after 24 h of incubation with fluorescently labeled free HMw-HA^Hylite Fluor 488^ and HMw-HA^Hylite Fluor 488^ -NPs. HA is labeled in these images in green, while the cell nucleus is labeled in blue. By comparing the two groups qualitatively, we observed that the intensity of the NPs inside the cells was higher than in the group treated with free HMw-HA, indicating that probably more NPs entered the cell compared to free HMw-HA.

Although the enhanced cellular uptake of NPs suggests a potential mechanism for activity, it remains unclear if cell internalization is necessary for HA biological effects. HA receptor-mediated interactions, such as binding to CD44, may be sufficient to activate signaling cascades that stimulate fibroblast activity and collagen synthesis, even without internalization.

Following, we examined whether the delivered HMw-HA, complexed as NPs, exhibits biological activity and stimulates collagen production.

Figure 4C presents confocal images of fibroblast cells treated for 72 h with non-labeled free HMw-HA and HMw-HA-NPs to assess the effect of their presence on collagen production. Collagen expression was revealed by indirect immunofluorescence collagen staining, as shown in green inside the cells. Quantification of these fluorescence intensities (Figure 4D) indicates that the addition of HMw-HA, both free and complexed, significantly increases the collagen production compared to the control. Furthermore, collagen production is higher when the HMw-HA was introduced as NPs with the Q-starch carrier. Monteiro et al. [6] examined the effect of HA on collagen production in fibroblast cells with and without the presence of the anti-CD44 antibody. When an anti-CD44 antibody was bound, there was a decrease in cell proliferation and collagen production, indicating that the CD44 receptor may be involved in the mechanism of stimulating collagen production. Our results (Figure 4D), demonstrating that the fluorescence intensity of collagen in cells treated with HMw-HA-NPs is higher compared to cells treated with free HMw-HA, can be explained by the assumption that free HMw-HA initially binds to CD44 receptors on the cell surface, limiting its further uptake or intracellular effects. However, over time (within a few hours), the free HMw-HA undergoes degradation, possibly due to the action of hyaluronidase enzymes present in the extracellular environment or secreted by fibroblasts. Previous studies have shown that the half-life of free HA in the skin can be as short as 1–2 days, and in some cases even a few hours, depending on its molecular weight and enzymatic activity [41]. In contrast, our experiments were conducted after 72 h of incubation, a period in which free HA is likely to have already been significantly degraded. This enzymatic activity likely reduces the availability of free HMw-HA to interact with the receptor over time. In contrast, HMw-HA in NP form is better protected, allowing for a slower release and, therefore, a more prolonged effect, which leads to sustained collagen production [42].

### 3.4. Preclinical Safety Study of HMw-HA-NPs

**Figure 5:**
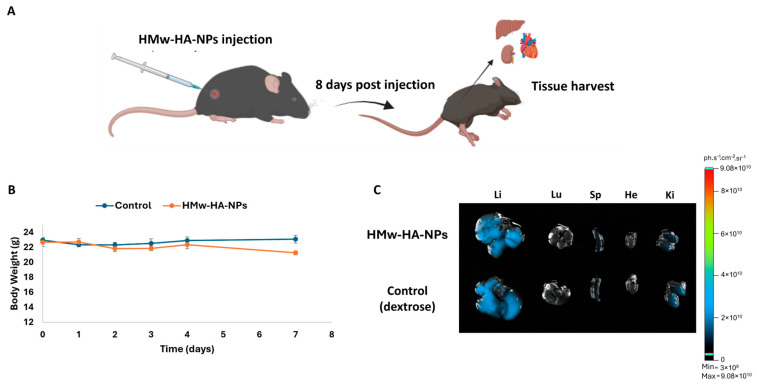
Preclinical safety study. (**A**) Illustration of the experimental setup; (**B**) C57BL\six mice body weight change at different time points post-intradermal injection of the following: 1. HMw-HA-NPs (N/O 0.25) (orange line), where Q-starch was labeled by 5-DTAF and HMw-Ha was labeled by Hylite Fluor 647 (50 μL, 260 mM of HA); and 2. control group, 5% dextrose (50 μL, blue line); average ± SEM (n = 4). (**C**) Representative fluorescence IVIS images of main organs, as follows: Li: liver; Lu: lungs; Sp: spleen; He: heart; Ki: kidneys, eight days post-HMw-HA-NPs intradermal injection.

The initial phase of any drug development process must involve preclinical safety assessment through animal testing to evaluate potential toxicity and establish a preliminary safety profile. Accordingly, we adopted a one-week observation window (eight days), which is routinely used in acute/sub-acute safety studies to capture accumulative effects and exceeds multiple cutaneous turnover cycles of native HA (half-life < 1 day), providing a conservative interval to reveal evolving toxicity under application-relevant dosing. For this purpose, Q-starch^5-DTAF^/HMw-HA^Hylite Fluor 647^ -NPs (50 μL, 260 mM of HA) were injected intradermally into C57BL\six mice (Figure 5A). As a control, sterile dextrose (5% in water) was also injected intradermally. Based on daily observations throughout the experiment, no mouse exhibited any passive behaviors such as hypopnea, tremors, or arching of the back, nor did they show any symptoms of poisoning such as loss of appetite, diarrhea, or vomiting up to eight days after injections. No significant differences in body weight were observed (Figure 5B), and all mice survived until they were sacrificed after the eight-day observation period. The major organs of the mice, including the heart, lungs, liver, spleen, and kidneys, were harvested after scarifice. Figure 5C presents their representative fluorescence images. Eight days post-injection, the NPs did not accumulate in any of the vital organs and probably did not penetrate the blood circulation, as there was no evidence of fluorescence signal in any of these organs responsible for substance clearance, primarily the liver and kidneys.

### 3.5. Residence Time and Biological Activity of HMw-HA-NPs In Vivo

**Figure 6:**
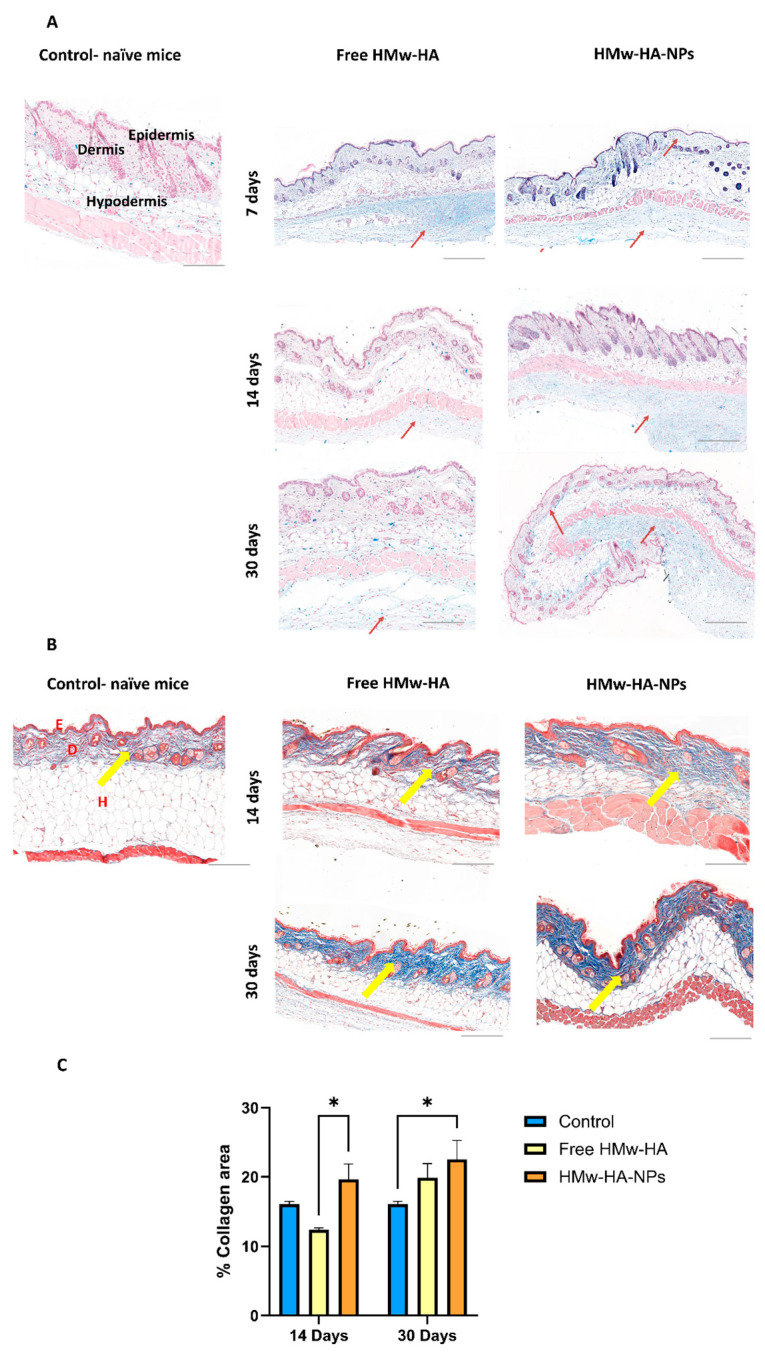
Representative histological images of skin section taken 14 and 30 days post-HMw-HA-NPs and free HMw-HA intradermal/subcutaneous injections. The same HA concentration (0.1% *w*/*v*) was injected into C57BL\six mice. The control group was naïve mice without treatment. (**A**) HA was stained with Alcian blue histochemical staining (light blue), red arrows show HA presence in the layer. (**B**) Collagen was stained with Masson’s trichrome histochemical staining (dark blue), yellow arrows show collagen presence in the layer. Bar scale indicates 200 μm, E—Epidermis, D—Dermis, and H—Hypodermis. (**C**) Collagen intensity quantification of histological skin section taken 14 and 30 days post-HMw-HA-NPs and free HMw-HA injections conducted using Qupath. (* *p* < 0.05) Average ± SEM (n = 4 for the control group; n = 8 for the free HMw-HA and HMw-HA-NPs).

After establishing a promising preclinical safety profile, we further investigated whether HMw-HA, when complexed to nano-sized particles with Q-starch as a carrier, exhibits a longer residence time (resists rapid clearance) within the skin. For this purpose, HMw-HA-NPs and free HMw-HA (as a positive control) at a volume of 25 μL were intradermally/subcutaneously injected with the same HMw-HA concentration (1 mg mL^−1^) into C57BL mice. After 7, 14, and 30 days, part of the mice were sacrificed, the skin at the injection area was removed, fixed in PFA, and sliced to a 5 μm thickness. Figure 6A presents representative histological skin sections at different time points post-injection, in which HA is stained in light blue, and the HA residence time in the skin tissues was estimated. In the images of the skin treated with free HMw-HA, we observe a light blue signal in the hypodermis, primarily below the dermis layer, seven days post-injection. As time progresses, this blue signal diminished at both 14 days and 30 days, consistent with the rapid cutaneous turnover of native HA driven by hyaluronidases (predominantly HYAL1/2), reported in human skin with typically <1 day, leading to faster clearance of free HA [43]. Conversely, skin treated with HMw-HA-NPs showed increased light blue coloration in the dermis and below the hypodermis up to 30 days post-injection (red arrows). This finding is in line with prior observations that organizing HA into supramolecular assemblies (e.g., fillers or HA-based nanoparticles) can reduce enzymatic accessibility and slow hyaluronidase-mediated degradation, thereby prolonging residence while preserving HA receptor interactions [44]. Our results can be explained by the association of HMw-HA with the Q-starch carrier, which probably protects the HMw-HA from rapid enzymatic degradation, allowing for a slower release, thus prolonging the residence time of HMw-HA within the skin.

Following these results, we examine whether the delivered HMw-HA is biologically active and can affect collagen production inside the skin. In contrast to our previous in vitro experiment with fibroblast cells, which focused on the cellular uptake and immediate effects of HMw-HA-NPs on collagen production over a short time frame, this study specifically investigates the effects of these NPs on skin tissue in a preclinical animal model over an extended period of time. After injecting HMw-HA-NPs and free HMw-HA, skin tissue sections were taken to detect collagen production using specific collagen staining. As can be seen in Figure 6B, there is an increase in collagen production, indicated by the dark blue coloration in the skin treated with HMw-HA-NPs compared to that treated with free HMw-HA (yellow arrows). Quantitative analysis of these images was conducted using QuPath software v0.5.0, which assessed collagen intensity relative to area, as shown on the Y-axis in Figure 6C. For collagen content estimation, we measured the area of blue color in Masson trichrome-stained slides. Briefly, the total sample area and blue area were segmented by thresholding the green channel of the RGB image and measured. The collagen area was calculated as the percentage of blue area out of the total sample area. A statistically significant difference can be observed between the HMw-HA-NPs and the free HMw-HA after 14 days. After 30 days, although there is no visible difference in collagen production between free HMw-HA and HMw-HA-NPs, the NPs does show a significant increase in collagen levels compared to the control.

By 30 days, both free HMw-HA and HMw-HA-NPs are likely degraded or eliminated from the tissue due to metabolic clearance, reaching a baseline level that no longer actively drives distinct collagen synthesis. The observed similarity between the free HMw-HA and NPs at this time point may reflect a return to homeostasis following the initial stimulatory effect. However, an additional explanation lies in the long half-life of collagen in the skin, which may lead to the accumulation of previously synthesized collagen that has not yet undergone degradation. We therefore hypothesize that extending the study period beyond 30 days would reveal a more pronounced difference between the groups, as the sustained release of HMw-HA from the nanoparticles continues to stimulate collagen production, whereas the free HA would have already been fully metabolized.

These findings suggest that HMw-HA, which forms NPs when complexed with Q-starch, remains biologically active for at least 30 days. This highlights the potential of our HMw-HA-NPs as a safe and long-lasting HMw-HA delivery system.

### 3.6. Skin Collagen Production: HMW-HA-NPs vs. HA Commercial Product

**Figure 7:**
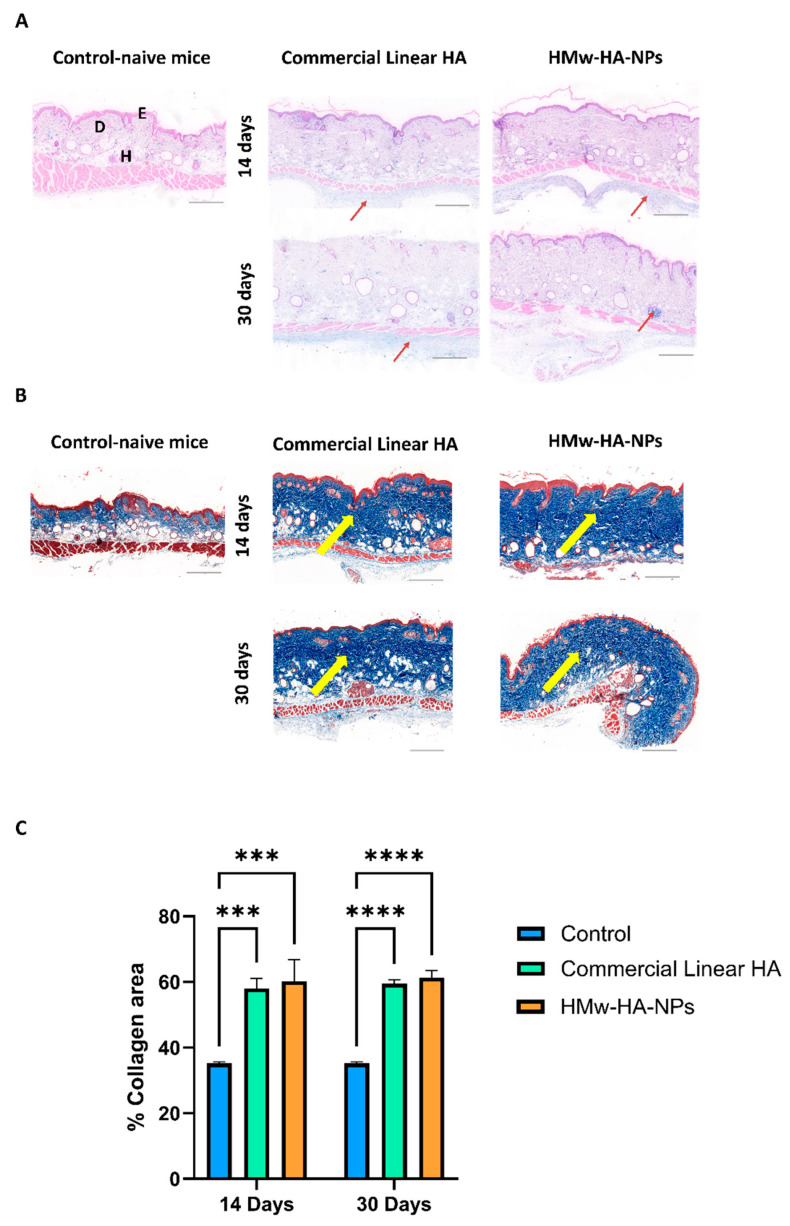
Representative histological images of skin section taken 14 and 30 days post-HMw-HA-NPs and commercial linear HA injections. The same HA concentration (14 mg mL^−1^) was injected dermally into Skh-1 hairless mice. (**A**) HA was stained with Alcian blue histochemical staining (light blue), red arrows show HA presence in the layer. (**B**) Collagen was stained with Masson’s trichrome histochemical staining (dark blue), yellow arrows show collagen presence in the layer. Bar scale indicates 200 μm, E—Epidermis, D—Dermis, and H—Hypodermis. (**C**) Collagen intensity quantification of histological skin section taken 14 and 30 days post-HMw-HA-NPs and free HMw-HA injections conducted using Qupath. (*** *p* < 0.001, **** *p* < 0.0001). Average ± SEM (n = 4 for all groups).

Since our NPs contain linear HMw-HA, we evaluated their performance in comparison to a commercial linear HA product (STYLAGE^®^ XXL of VIVACY Paris, no indication regarding its molecular weight). This product is used as a skin booster to rejuvenate the skin by restoring moisture and filling in fine surface wrinkles. We injected our HMw-HA-NPs with an HMw-HA concentration of 14 mg mL^−1^, which is 14 times higher than in our previous experiment (matching the commercial linear HA recommended concentration according to the manufacturer), into the dermis/subcutaneous layer of Skh-1 hairless mice, suitable for the evaluation of skin appearance. Figure 7A shows representative histological skin sections at different time points post-injection, in which the HMw-HA is stained in light blue. As can be seen, after 14 and 30 days, HA was present in both experimental groups, approximately at the same level. We continued to test the amount of collagen at 14 and 30 days post-injection. As shown qualitatively in Figure 7B, the intensity of the dark blue stain, which indicates collagen fibers, appears to be similar between the two treatment groups but differs from that of the control group, which did not undergo any treatment. The quantification results (Figure 7C) show significant statistical differences between the treated groups and the untreated control skin. Specifically, the collagen levels in the skin injected with HMw-HA-NPs are similar to those observed with commercial linear HA injections, both significantly higher than the collagen levels in untreated skin.

Consequently, these results suggest that our formulation has the potential to compete with commercially available linear HA in terms of collagen production. Importantly, the key benefit of the NP platform is not only in its performance under injection but also in its design for a future non-invasive route: HMw-HA-NPs are intended to be delivered topically with low-frequency ultrasound (LFUS), potentially enabling a needle-free regimen that addresses pain, injection-related adverse events, and clinic-dependent procedures. Furthermore, our HMw-HA-NPs probably offer additional advantages over the linear commercial HA. Specifically, our system is designed for sustained release, which suggests that over a longer testing period, it may provide prolonged activity compared to free HA, potentially enhancing collagen production over time. While extended time points are beyond the scope of the present study, the stability data (morphology, ζ-potential, viscosity) support the feasibility of sustained HA availability from the NP formulation. Moreover, while the molecular weight of the commercial HA is not explicitly stated, we know from the manufacturer that it includes lower-molecular-weight fractions to enable injections. In contrast, our NPs consist entirely of very high-molecular-weight HA, ensuring that the entire formulation benefits from the superior efficacy of larger HA molecules while still maintaining injectability. Taken together, equivalence under injection coupled with the prospect of LFUS-mediated, needle-free delivery underscores the translational value of the NP approach.

As previously mentioned, the Q-starch component in the NPs condenses and reduces the size of HMw-HA to a nanoscale size, in contrast to linear commercial HA, which is probably larger and more dispersed. This nanoscale size of the HMw-HA-NPs is particularly critical for advancing to the next step, where we aim to assess the penetration of HMw-HA in NP form, using a non-invasive US-mediated delivery approach. This non-invasive value proposition is central to our strategy: the NP structure is crucial for this approach, as smaller particle size greatly enhances its ability to penetrate the skin efficiently when coupled with LFUS-assisted sonophoresis.

### 3.7. A Non-Invasive US-Mediated Dermal Penetration Approach (In Vivo Study) 

A major drawback of injecting commercial HA as a skin rejuvenation product is the need for multiple injections across large areas of the face, which can be both time-consuming and extremely painful for the patient. Encouraged by our results showing that the HMw-HA-NPs performance in terms of safety and collagen production is comparable to that of commercially available linear HA, we aimed to further investigate a non-invasive delivery approach for these HMw-HA-NPs.

**Figure 8:**
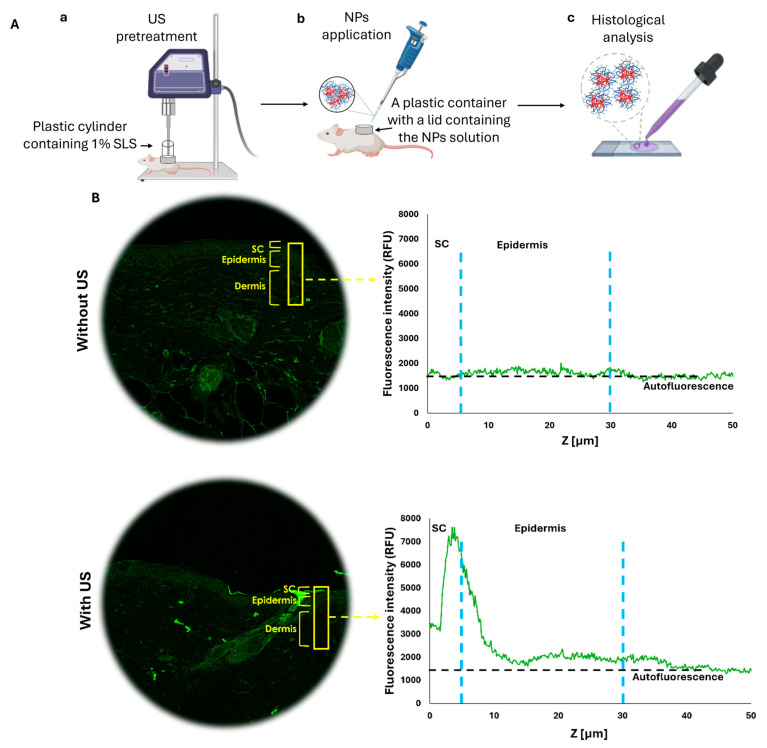

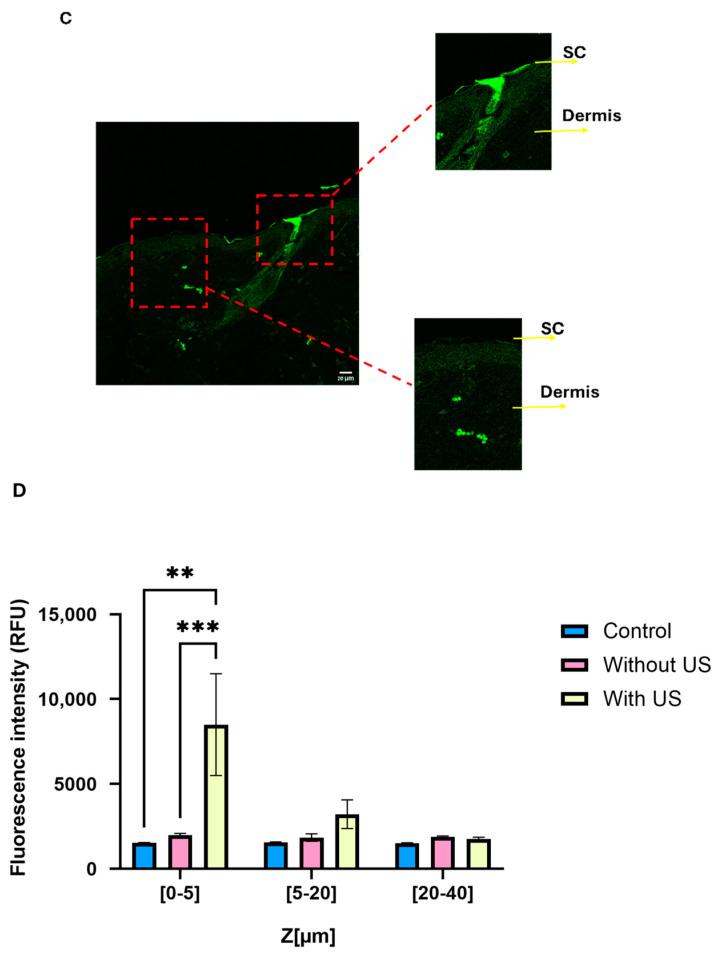
(**A**) In vivo experimental setup: (**a**) US pre-treatment (20 KHz, 12 W/cm^2^, 59% duty cycle), (**b**) topical application of HMw-HA-NPs (260 mM), and (**c**) histological analysis of Q-starch^5-DTAF^/HMw-HA-NPs. (**B**) Representative confocal images of mice skin cross-sections (left) and fluorescence intensity quantification of Q-starch^5-DTAF^ (right) after 24 h of incubation with Q-starch^5-DTAF^/HMw-HA-NPs (calculated by Image J). (**C**) An enlarged fluorescent image of skin pre-treated with US, representing two different areas of HMw-HA-NPs penetration. (**D**) Fluorescence intensity quantification in histological skin sections taken 24 h post Q-starch^5-DTAF^/HMw-HA-NPs administration using Image J. (** *p* < 0.01, *** *p* < 0.001). Average ± SEM (n = 4 for all groups).

In the next step, we aimed to evaluate whether US pre-treatment can enhance the penetration of topically applied HMw-HA-NPs, providing an effective alternative to traditional injection-based delivery methods. We examined the effects of US treatment using a specific setup for our in vivo model, as illustrated in Figure 8A. Following the US pre-treatment (20 KHz, 12 W/cm^2^, 50% duty cycle), HMw-HA-NPs were applied topically to the US-exposed area on the dorsal skin of Skh-1 mice, as detailed in the Materials and Methods section. Higher concentrations rendered the preparation too viscous for uniform spreading and coupling and were not compatible with LFUS-assisted delivery; therefore, 0.1 mg mL^−1^ was adopted across in vitro and in vivo studies. A total of 24 h following the application, the mice were sacrificed, and their skin was carefully excised. The excised skin was washed to remove any remaining HMW-HA-NPs on the surface and subsequently sectioned into 5 µm slides for analysis. Using confocal microscopy, we measured the fluorescence intensity (using ImageJ software) of the HMw-HA-NPs from the SC to a depth of 50 μm in the skin, focusing on the regions marked by yellow rectangles (Figure 8B). As shown in Figure 7B, pre-treatment with US led to a noticeable fluorescence intensity in the SC layer and the upper layers of the viable epidermis, indicating penetration and accumulation of HMw-HA-NPs within this region. In contrast, skin samples that were not exposed to US pre-treatment exhibited fluorescence intensities at the level of the autofluorescence of the mouse skin. This suggests that without US pre-treatment, the HMw-HA-NPs did not penetrate the skin, as the detected signal corresponds to background fluorescence. In line with these findings, topical free HMw-HA failed to penetrate ex vivo porcine skin under our LFUS settings (Supplementary Figure S1). Accordingly, we did not include an in vivo group treated with free HMw-HA and LFUS, since without ex vivo penetration, such a comparison was unlikely to yield meaningful information.

Figure 8C presents a magnification of the image in Figure 8B with US pre-treatment. The two areas that are enlarged (marked with red squares) reveal that the US exposure is not homogeneous across the entire skin, with some regions being more affected by the exposure. In one area, there is an accumulation of HMw-HA-NPs in the SC (on the top right), while in another area there are no HMw-HA-NPs in the SC at all, with some located in a deeper layer, the dermis (bottom left). Therefore, for quantification of these results, three different skin samples were taken from each mouse. From each skin sample, three random rectangles (marked in yellow) were examined and quantified to assess the difference in the fluorescence intensity of the HMw-HA-NPs in the three groups. The quantification results are presented in Figure 8D. Skin pre-treated with US exhibited significantly higher fluorescence intensity in the SC compared to untreated samples, confirming the enhanced penetration of HMw-HA-NPs. In contrast, samples without US pre-treatment showed no significant difference in fluorescence intensity between those treated with NPs and control groups, indicating that HMw-HA-NPs did not penetrate the skin. These results highlight the efficacy of combining US pre-treatment with the nanoparticle carrier system in facilitating deeper skin penetration of HMw-HA-NPs.

### 3.8. Biological Activity of HMw-HA-NPs After US Pre-Treatment

**Figure 9:**
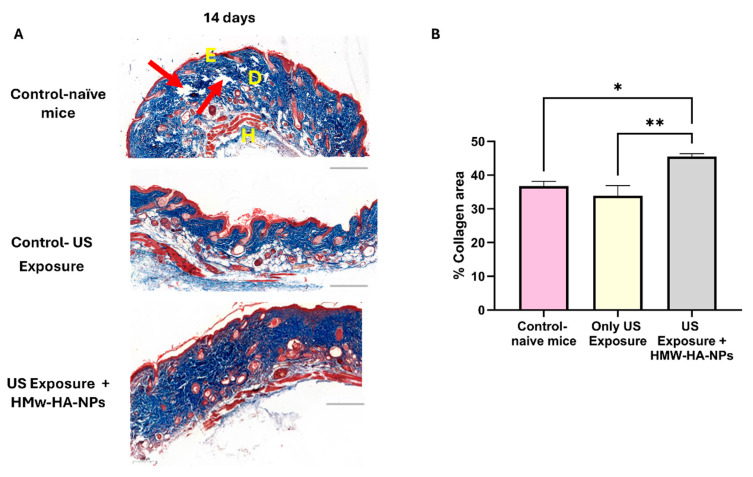
(**A**) Representative images of histological skin sections taken 14 days post different treatments: control group (without any treatment); only US exposure (without HMw-HA-NPs administration); HMw-HA-NPs (260 mM) topical administration after US pre-treatment. Collagen was stained with Masson’s trichrome histochemical staining (dark blue), red arrows show collagen presence in the layer. Bar scale indicates 200 μm, E—Epidermis, D—Dermis, and H—Hypodermis. (**B**) Collagen intensity quantification of histological skin section taken 14 and 30 days post-HMw-HA-NPs topical administration conducted using Qupath. (* *p* < 0.05, ** *p* < 0.01). Average ± SEM (n = 4 for all groups).

To determine whether the HMw-HA-NPs that penetrated the skin post-US treatment remained biologically active and stimulated collagen production, we conducted an experiment assessing collagen levels 14 days after US pre-treatment followed by topical application of HMw-HA-NPs. Figure 9A presents representative panoramic microscopy images of mouse skin, indicating that HMw-HA-NPs maintain their biological activity when delivered after US exposure, as evidenced by enhanced collagen production. This increase is observed as more compact and densely arranged collagen fibers in the dermis layer (indicated by the enhanced blue staining). In contrast, control samples exhibit lower collagen density and visible gaps between fibers (red arrows). Quantitative image analysis (Figure 9B) further supports these observations. A statistically significant increase in collagen intensity is observed in the group receiving US pre-treatment followed by HMw-HA-NPs application compared to control groups after a 14-day period. These findings suggest that HMw-HA-NPs may provide therapeutic potential for enhancing collagen synthesis following US-assisted delivery.

Importantly, the magnitude of the collagen response achieved with the non-invasive route is substantial: under US-assisted topical delivery, we observed ~40% collagen area at day 14, compared with ~60% in the injected setting (Figure 7), even though the injected condition deposits the entire dose directly into the dermis, while the topical US approach delivers only a fraction of the surface-applied dose (with part remaining on the surface and being washed off). Thus, the topical US-NPs regimen attains a large fraction of the “injection-level” collagen outcome without intradermal deposition, highlighting its translational value as a needle-free alternative.

We hypothesize that this enhanced collagen production in the dermis, despite limited penetration of HMw-HA-NPs beyond 5 microns, may be due to a cascade effect initiated in the upper layers of the skin. Consistent with published evidence, HA engagement of CD44/RHAMM on epidermal/upper-dermal cells activates ERK and PI3K/AKT pathways and releases paracrine cues that stimulate dermal fibroblasts to synthesize collagen, even when the primary stimulus is superficial [45,46]. It is possible that the HMw-HA present on the surface of the NPs, or gradually released from them, stimulates cells in the superficial layers, triggering a signaling cascade that extends to the deeper dermal layers. This biological response could lead to increased collagen production by fibroblasts in the dermis, even without direct contact with the NPs. Importantly, these findings were observed following only a single US pre-treatment and one-time application of HMw-HA-NPs, emphasizing the durability and therapeutic promise of this strategy over a prolonged period for enhancing collagen production in dermal tissues.

## 4. Conclusions

**Figure 10:**
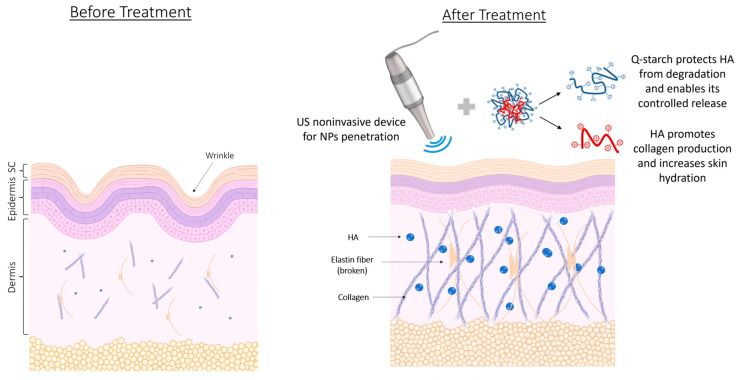
Schematic skin structure before and after treatment with US non-invasive device and HMw-HA-NPs. Created by Biorender.

This study elucidates the efficacy of NPs containing linear HMw-HA combined with Q-starch as a novel dermal delivery system. The NPs demonstrated several advantageous properties, including sustained efficacy, enhanced biological activity, resistance to enzymatic degradation, and enhanced chemical stability at high concentrations, ensuring that the HA remained intact and functional without degradation. Moreover, the study highlighted the potential for the non-invasive delivery of the HMw-HA-NPs, as the combination of US pre-treatment and HMw-HA-NPs led to significantly greater dermal penetration and collagen production compared to either treatment alone (Figure 10). This enhanced penetration was corroborated through in vivo experiments, where the combination of US and NPs resulted in effective dermal delivery. The primary advantage of this system lies in its ability to deliver HMw-HA non-invasively into deeper skin layers while maintaining its biological activity over prolonged periods, thus offering a potential paradigm shift in dermal drug delivery and regenerative skin therapies.

## Data Availability

The original contributions presented in this study are included in the article/Supplementary Material. Further inquiries can be directed to the corresponding author.

## Supplementary Materials

The following supporting information can be downloaded at: https://www.mdpi.com/article/10.3390/nano15221739/s1. Figure S1: Representative confocal images of porcine ear skin cross section after 24 h of incubation with 0.1% wt. HAHylite Fluor 647 after US pre-treatment. Left—bright field confocal image. Right—represent pixel fluorescence intensity of the HAHylite Fluor 647 complexes as a function of the distance from SC, up to a depth of 200 μm, calculated by Image J. HA labeled in red. (Bar = 20 μm). The blue lines represent a separation between the layers, as follows: between the SC and epidermis at 20 μm, between the epidermis and dermis at 100 μm, and dermis layer from 100 μm up to 200 μm. The black dashed line describes the skin’s autofluorescence at the wavelength of the labeled HA.
