# Supplementary material for: Advancing Skin Rejuvenation Through Ultrasound-Enhanced Non-Invasive Delivery of Hyaluronic Acid Nanoparticles
Source: Nanomaterials (Basel). 2025 Nov 18;15(22):1739. doi: 10.3390/nano15221739 (PMC12655556; doi:10.3390/nano15221739)
Supplement: Supplementary file 1 [file nanomaterials-15-01739-s001.zip › nanomaterials-3966399-supplementary.pdf]

Supporting Information

1. Figures

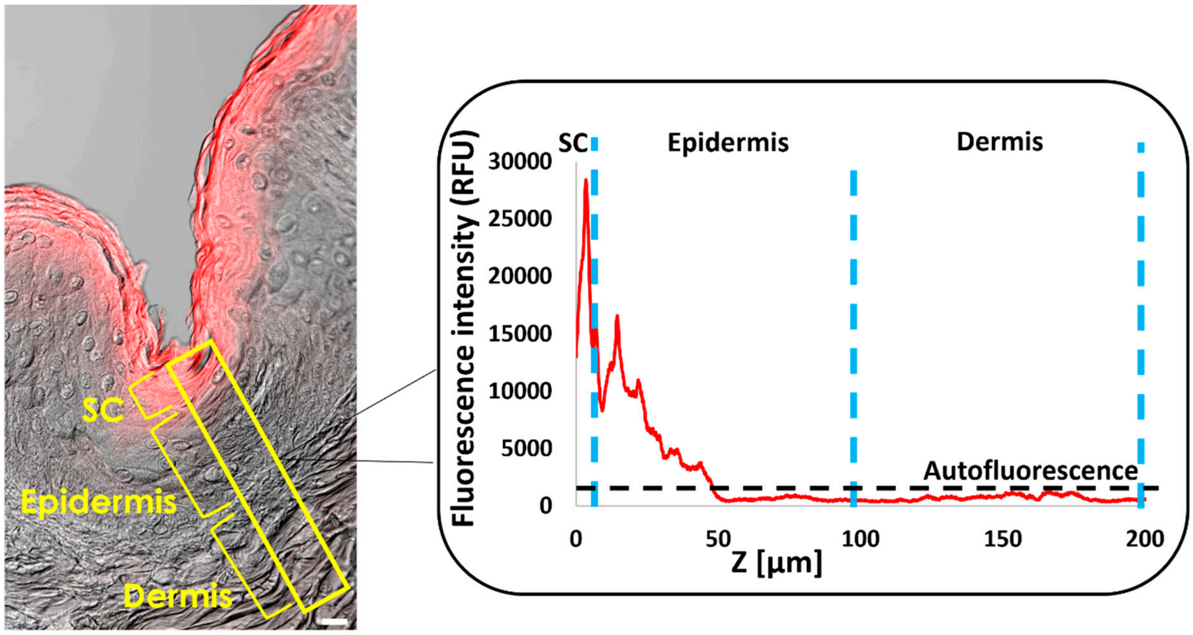

Figure S1. Representative confocal images of porcine ear skin cross-section after 24 h of incubation with 0.1% wt. HA$^{Hylite\ Fluor\ 647}$ after US pretreatment. Left- bright field confocal image. Right- represent pixel fluorescence intensity of the HA$^{Hylite\ Fluor\ 647}$ complexes as a function of the distance from SC, up to a depth of 200 µm, calculated by Image j. HA labelled in red. (Bar= 20 µm). The blue lines represent a separation between the layers: between the SC and epidermis at 20 µm, between the epidermis and dermis at 100 µm, and dermis layer from 100 µm up to 200 µm. The black dashed line describes the skin's autofluorescence at the wavelength of the labeled HA.
